# Analysis of “visible in retrospect” to monitor false-negative findings in radiological reports

**DOI:** 10.1007/s11604-022-01338-2

**Published:** 2022-09-19

**Authors:** Tomoyuki Noguchi, Koji Yamashita, Shuji Matsuura, Ryotaro Kamei, Junki Maehara, Kiyomi Furuya, Shino Harada, Saki Adachi, Yasushi Okada

**Affiliations:** 1 Department of Radiology, National Hospital Organization Kyushu Medical Center, 1-8-1 Jigyohama, Chuo-ku, Fukuoka City, Fukuoka Province 810-8563 Japan; 2 Department of Clinical Research, National Hospital Organization Kyushu Medical Center, 1-8-1 Jigyohama, Chuo-ku, Fukuoka City, Fukuoka Province 810-8563 Japan; 3 Medical Safety Management Unit, National Hospital Organization Kyushu Medical Center, 1-8-1 Jigyohama, Chuo-ku, Fukuoka City, Fukuoka Province 810-8563 Japan; 4 Education and Training Office, Department of Clinical Research, Center for Clinical Sciences, National Center for Global Health and Medicine, 1-21-1 Toyama, Shinjuku-ku, Tokyo, 162-8655 Japan

**Keywords:** False-negative finding, Missed diagnosis, Radiology, Visible in retrospect, Radiological report

## Abstract

**Purpose:**

False-negative findings in radiological reports can lead to serious adverse patient outcomes. We determined the frequency and tendency of false-negative findings in radiological reports by searching for words related to “visible in retrospect”.

**Methods:**

In the period of 34 months, we extracted radiological reports containing words related to “visible in retrospect”. Of these reports, we extracted false-negative findings that were not described in past reports and were first detected retrospectively. Misinterpretations were excluded. The occurrences of the terms that we identified were analyzed by all examinations, modality, month, and anatomical and lesion classifications were analyzed.

**Results:**

Of the 135,251 examinations, 941 reports (0.71%) with 962 findings were detected, with an average of 1.4 findings per business day. By modality, 713 of 81,899 (0.87%) CT examinations, 208 of 36,174 (0.57%) MR, 34 of 9,585 (0.35%) FDG-PET-CT, 2 of 2,258 (0.09%) digital radiography, and 5 of 5,335 (0.09%) other nuclear medicine examinations were found. By anatomical classification, there were 383 (40%) in chest, 353 (37%) in abdomen, 162 (17%) in head, 42 (4.4%) in face and neck, 9 (0.93%) in extremity, and 13 (1.4%) in others. By lesion classification, we identified 665 (69%) for localized lesion, 170 (18%) for vascular lesion, 83 (8.6%) for inflammatory lesion, 14 (1.5%) for traumatic lesion, 12 (1.2%) for organ dysfunction, 11 (1.1%) for degenerative lesion, and 7 (0.7%) for the others. Notable high-frequency specific site diseases by modality were 210 (22%) of localized lesions in lung on CT.

**Conclusion:**

Our results demonstrated that missed lung localized lesions on CT, which account for about a fifth of false-negative findings, were the most common false-negative finding.

## Introduction

False-negative errors account for 0.8–5% of daily clinical diagnostic imaging errors, and false-negative error rates at 13–90% were recorded under experimental conditions used to measure radiologists’ diagnostic ability [1–4]. A false-negative finding, or missed diagnosis, was the most common type of findings in these studies; for example, of the 12 subgroups of radiological error types defined by Kim and Mansfield, the false-negative finding accounted for 42% [5]. False-negative findings in an imaging diagnosis can lead to serious patient outcomes, as they can result in a delayed diagnosis and/or delayed treatment.

Most cases of false-negative findings are retrospectively detected by a follow-up examination. Findings that are visible in retrospect but were present in a previous examination are usually included in the radiological report of the most recent examination. However, an interpretation’s oversight is sometimes identified by a second reading of an imaging examination by a clinician or another radiologist or in a patient review meeting. In such cases, the revision of a finding might be added to the radiological report as an addendum. This is considered an example of “findings visible in retrospect,” in a sense. In such cases, the false-negative findings are likely to be included in the radiological report. We speculated that false-negative findings might be simply and easily extracted by searching radiological-report databases for words or phrases related to “visible in retrospect” in the radiological reports. We conducted the present study to evaluate the false-negative findings obtained by a search for words and phrases related to “visible in retrospect” in radiological reports.

## Methods

### Study design

This retrospective analysis was approved by the Ethics Review Committee of our hospital, waiving the need for written informed consent from the patients (No. 21C144).

### Extraction of false-negative findings in radiological reports

We analyzed a total of 135,251 of radiological reports made at our over 700-bed hospital with over 40 departments during the 34 month period from October 2018 to July 2021. These reports included 81,899 reports of computed tomography (CT) findings, 36,174 of magnetic resonance imaging (MR), 9,585 of digital radiography (DR), 2,258 of positron emission tomography and computed tomography using ^18^F-fluorodeoxyglucose (PET), and 5,335 of other nuclear medicine (NM) examinations. We extracted all radiological reports containing wording related to “visible in retrospect:” ‘looking back,’ ‘reviewing back,’ ‘retrospective,’ ‘re-reading,’ ‘correction,’ ‘amendment,’ or ‘addendum.’ We then performed the same wording search in the content of each of extracted radiological report to identify the details of false-negative findings. Misinterpretations of findings were excluded, and false-negative findings that were not mentioned in previous reports were finally registered.

### Number and qualification of radiologist

Different personnel had created the radiological reports in our hospital’s diagnostic radiology department over the study period: from October 2018 to March 2020, the reports were by ten full-time board-certified diagnostic radiologists and two residents; from April 2020 to May 2020, the reports were by five full-time board-certified diagnostic radiologists; and from June 2020 to July 2021, the reports were made by six full-time radiologists. At least two board-certified specialists for nuclear medicine were included through the study period. At least one expert with more than 10 years of neuroradiology experience was included from April 2020 to July 2021. There were 120,732 examinations (89%) for which imaging reports were completed within 2 days regardless of weekdays or holidays.

### Regional organ classification (ROC)

**Fig. 1:**
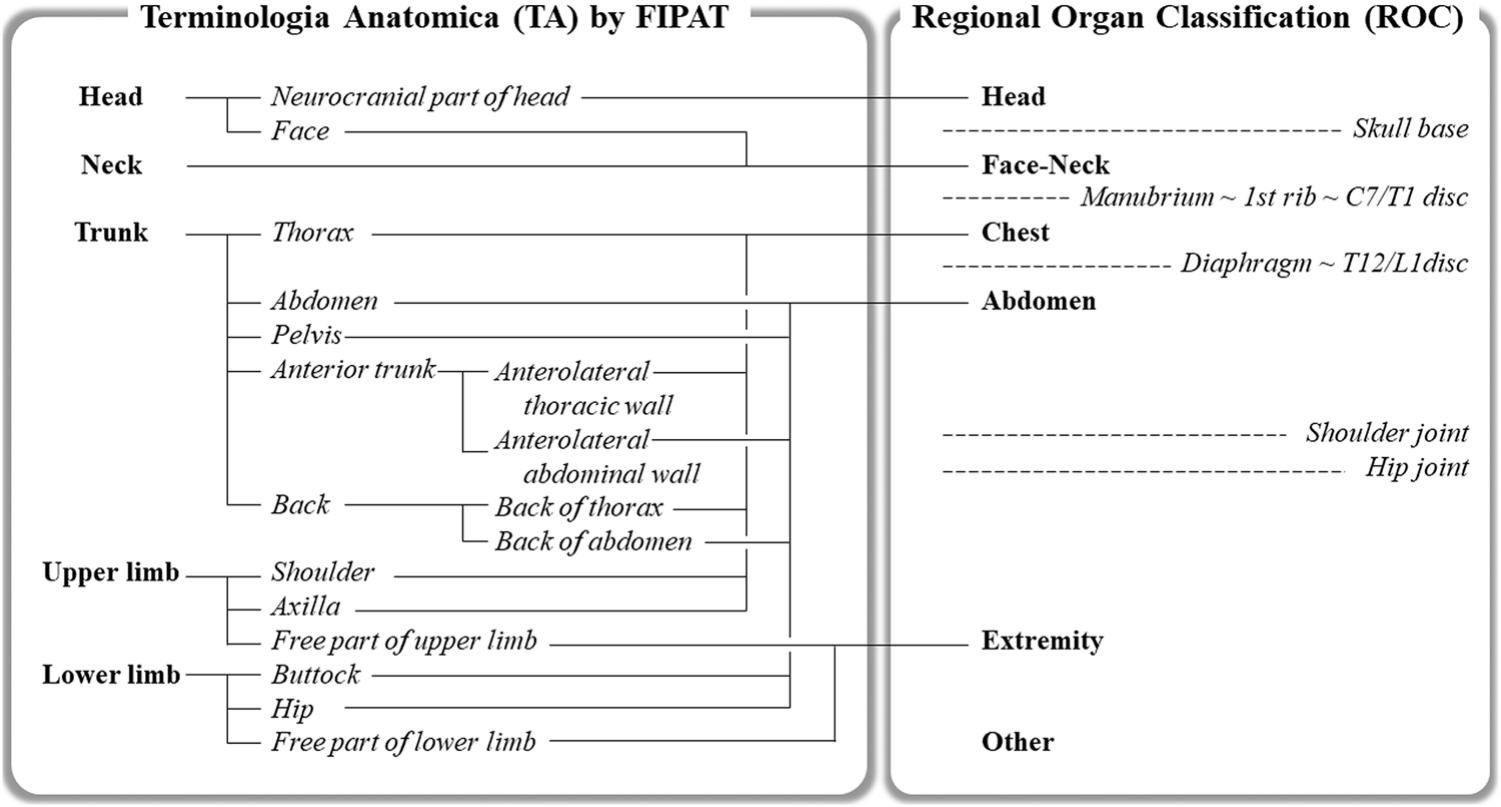
Reassignment of the *Terminologia Anatomica* categories to the Regional Organ Classification (ROC). The *Terminologia Anatomica* (TA) developed by the Federative International Programme for Anatomical Terminology (FIPAT) describes parts of the human body as ‘Head,’ ‘Neck,’ ‘Trunk,’ ‘Upper Limb,’ and ‘Lower Limb’ in the General Anatomy chapter; we re-classified these parts to six major categories in the ROC: Head, Face–Neck, Chest, Abdomen, Extremity, and Other. The categories' boundaries are the skull base, the manubrium—1st rib—C7/T1 disc, the diaphragm—T12/L1disc, the shoulder joint, and the hip joint. A false-negative finding extending equally to multiple regions was assigned to other of the ROC

The publication *Terminologia Anatomica* (hereinafter abbreviated as the “TA”), which is the international standard for human anatomical terminology developed by the Federative International Programme for Anatomical Terminology (FIPAT), provides a systemic organ classification [6]. However, findings obtained medical imaging modalities such as CT and MR are usually of a part of the body that is separated simply by a plane perpendicular to the long axis of the body. We therefore created a Regional Organ Classification (hereinafter abbreviated as the “ROC”) which was adjusted for medical images based on the TA. In its first chapter (General Anatomy), the TA describes parts of human body as the ‘Head,’ ‘Neck,’ ‘Trunk,’ ‘Upper Limb,’ and ‘Lower Limb.’ To reclassify these terms, we defined six major ROC categories: Head, Face-Neck, Chest, Abdomen, Extremity, and Other. Figure 1 illustrates the matching concept of the TA and the ROC and the details were described Appendix. For each of the six major ROC categories, we divided the minor category of the ROC into five parts: organ, vessel, lymph node, membranous structure forming cavity, and bone & soft tissue. Table 1 provides the details of the ROC’s minor category.

**Table 1:** Major and minor categories of the Regional Organ Classification (ROC)

Major category	Minor categories
Head	Brain, Head vessel, Meninx, Head bone & soft tissue
Face-neck	Eyes, Nose, Pharynx, Larynx, Saliva, Thyroid, Cervical trachea, Cervical esophagus, Face–Neck vessel, Face–Neck lymph node, Face–Neck bone & soft tissue
Chest	Lung, Breast, Heart, Mediastinal trachea, Thoracic esophagus, Chest vessel, Chest lymph node, Pleura, Chest bone & soft tissue
Abdomen	Liver, Pancreas, Gastrointestine, Biliary system, Urinary system, Adrenal gland, Spleen, Female genital organ, Male genital organ, Abdominal vessel, Abdominal lymph node, Peritoneum, Abdominal bone & soft tissue
Extremity	Upper extremity, Lower extremity
Other	Whole body bone

### Lesion classification

We extracted the suspected diseases in these radiological reports or imaging findings and assigned them into six categories: Localized Lesion, Vascular Lesion, Inflammatory Lesion, Traumatic Lesion, Organ Dysfunction, Degenerative Lesion, and Other. False-negative findings were mainly assigned to the specific lesion classification based on the contents of the radiological report, and if this was quite difficult to determine, the judgment was made with reference to the patient’s electronic medical records. Table 2 explains the six lesion classifications.

**Table 2:** Lesion classification

Lesion classification	Items
Localized lesion	Patchy opacity, lymphoid lesion, malignant lesion, low attenuation, cystic lesion, solid lesion, etc.
Vascular lesion	Vascular dissection, vasculitis, vascular malformation, hematoma, blood, infarction, bleeding, etc.
Traumatic lesion	Fracture, other trauma, etc.
Organ dysfunction	Liver function, splenomegaly, atrophy, etc.
Degenerative lesion	Wallerian degeneration, bronchiectasis, arthropathy, myelomalacia, etc.
Other	Zenker's diverticulum, expansion of the renal pelvis with unknown etiology, gas in the aorta with unknown etiology, spontaneous pneumothorax, etc.

### Statistical analysis

Descriptive statistics were performed, including the incidence of false-negative findings by examination, time period, modality, ROC category, and lesion classifications.

## Results

Of total of 135,251 examinations, we identified 940 reports (0.70%) and 962 findings (0.71%; 21 reports containing two findings and a single report containing three findings), with an average of 28 findings per month and 1.4 findings per work day (Table 3).

**Table 3:** The incidence of false-negative findings by examination and time period

Item	Value
Total examination	135,251
CT/MR/DR/PET/NM	81,899/36,174/9,585/2,258/5,335
No. of radiological reports with false-negative findings	940
No. of false-negative findings	962
One finding/2 findings/3 findings in a report	917/21/1
Incidence rate per examination	0.71% per exam
Incidence rate per month	28/month
Incidence rate per business day over 684 days	1.4/day

Table 4 demonstrates the number of false-negative findings by modality and major ROCs: Of the 962 false-negative findings, approximately three-quarters were CT (74%), one-fifth was MR (22%), and the others were found in PET (4%), NM (1%), and DR (0.2%). In the major ROC categories, two-fifths were Chest (40%) and Abdomen (37%), one-sixth were Head (17%), and the others were found in Face- Neck (4%), Extremity (1%), and Others (1%). The appreciable frequent major ROCs by modality were Chest in CT (37%) and Head in MR (11%).

**Table 4:** Number of false-negative findings by modality and major ROCs

Modality	Head	Face–neck	Chest	Abdomen	Extremity	Others	Total
CT	57 (6%)	32 (3%)	353 (37%)	256 (27%)	4 (0.4%)	11 (1%)	713 (74%)
MR	105 (11%)	7 (1%)	18 (2%)	73 (8%)	3 (0.3%)	2 (0.2%)	208 (22%)
PET	0	3 (0.3%)	10 (1%)	20 (2%)	1 (0.1%)	0	34 (4%)
NM	0	0	0	4 (0.4%)	1 (0.1%)	0	5 (1%)
DR	0	0	2 (0.2%)	0	0	0	2 (0.2%)
Total	162 (17%)	42 (4%)	383 (40%)	353 (37%)	9 (1%)	13 (1%)	962

Table 5 shows the number of false-negative findings by minor ROCs: Among the minor ROC categories, false-negative findings were the most common in Lung (27%), followed by Liver (11%) and Brain (9%).

**Table 5:** Number of false-negative findings by minor and major ROCs

Minor ROC	Head	Face–neck	Chest	Abdomen	Extremity	Others	Total
Organ	Brain: 83 (9%)	Face–neck organ: 24 (2%)	Lung: 257 (27%)	Liver: 105 (11%)	Lower extremity: 6 (1%)	Whole body bone: 13 (1%)	660 (69%)
			Breast: 22 (2%)	Pancreas: 40 (4%)			
			Thoracic esophagus: 4 (0.4%)	Gastrointestine: 31 (3%)	Upper extremity: 3 (0.3%)		
				Urinary system: 20 (2%)			
			Heart: 1 (0.1%)	Biliary system: 19 (2%)			
				Adrenal glands: 13 (1%)			
				Spleen: 12 (1%)			
				Female genital organ: 4 (0.4%)			
				Male genital organ: 3 (0.3%)			
Vessel (V)	Head V: 53 (6%)	Face–neck V: 4 (0.4%)	Chest V: 20 (2%)	Abdominal V: 15 (2%)			92 (10%)
Lymph node (LN)		Face–neck LN: 3 (0.3%)	Chest LN: 25 (3%)	Abdominal LN: 28 (3%)			56 (6%)
Membranous structure forming cavity	Meninx: 21 (2%)		Pleura: 13 (1%)	Peritoneum: 28 (3%)			62 (6%)
Bone & soft tissue (BS)	Head BS: 5 (1%)	Face-neck BS: 11 (1%)	Chest BS: 41 (4%)	Abdominal BS: 35 (4%)			92 (10%)

Table 6 shows the number of false-negative findings by lesion classification and major ROCs: According to the lesion classification, two-thirds were a localized lesion (69%), one–six were a vascular lesion (18%), and less than 10% each were found in inflammatory lesion (9%), traumatic lesion (1%), organ dysfunction (1%), degenerative lesion (1%), and seven of other (1%). The most common major ROCs of the lesion category were chest localized lesions (32%), followed by head vascular lesions (11%), chest inflammatory lesions (4%).

**Table 6:** Number of false-negative findings by lesion classification and major ROCs

Lesion classification	Head	Face-neck	Chest	Abdomen	Extremity	Others	Total
Localized lesion	46 (5%)	25 (3%)	308 (32%)	271 (28%)	4 (0.4%)	11 (1%)	665 (69%)
Vascular lesion	106 (11%)	7 (1%)	22 (2%)	30 (3%)	5 (1%)	0	170 (18%)
Inflammatory lesion	2 (0.2%)	3 (0.3%)	40 (4%)	37 (4%)	0	1 (0.1%)	83 (9%)
Traumatic lesion	0	1 (0.1%)	6 (1%)	7 (1%)	0	0	14 (1%)
Organ dysfunction	3 (0.3%)	2 (0.2%)	1 (0.1%)	6 (1%)	5 (1%)	0	12 (1%)
Degenerative lesion	5 (1%)	2 (0.2%)	4 (0.4%)	0	0	0	11 (1%)
Others	0	2 (0.2%)	2 (0.2%)	2 (0.2%)	0	1 (0.1%)	7 (1%)

The notable high-frequency false-negative findings in the combination of modality, the major ROC, and the lesion classification concerned a localized lesion in a lung on CT (*n* = 210, 22%), followed by a localized lesion in the liver on CT (*n* = 62, 6%), and a vascular lesion in a head vessel on MR (*n* = 45, 8%).

## Discussion

False-negative error is one of the most critical issues in diagnostic radiology. Earlier reports have referred to false-negative errors as a perceptual error, non-identification error, missed diagnosis, omission error, underreading error, overlooking error, or oversight error [1–5, 7–12]. False-negative error has also been called “delayed diagnosis”, since it is identified later than the initial diagnosis. “Diagnostic discrepancy” or “diagnostic disagreement” is used as an indirect term, because the finding was deemed negative in the first reading and positive in a second reading. The description that a finding is “visible in retrospect” is used, because many such findings are discovered by looking at past imaging examinations with radiological reports based on the most current imaging inspection.

False-negative errors are unfortunately common in radiology practice, as is also true in the other clinical departments [2, 13–15]. Serious false-negative errors in medicine are unacceptable to the general public. The same legal penalties as those imposed for serious and unavoidable traffic accidents have been proposed for false-negative errors by radiologists [9]. Therefore, false-negative errors should be controlled and potentially eliminated by continuous monitoring and analyses and by taking steps to avoid causing medical distrust among the public.

Lee proposed that methods that could be applied for successful quality management in radiology must be reliable, robust, consistent, and easy to follow [10]. However, most patients undergoing imaging examinations do not have a pathological diagnosis or genetic confirmation, and it is thus difficult to extract definitive false-negative findings. Methods that have been suggested include auditing by the random pick-up of a small number of cases [10], validation in autopsy cases [16], radiology discrepancy meetings [5, 17, 18], double reading [19, 20], and an error registration system [11, 21–23]. While these methods are effective in that their use will enable medical personnel to identify errors more reliably and will also serve as error education, such errors are likely not representative of those occurring in hospitals on a daily basis [4]. In addition, these methods require extra actions in addition to normal reporting work, e.g., to introduce a new safety system or perform case sampling for auditing.

Diaz et al. analyzed the frequency of diagnostic errors in radiological reports by extracting radiological reports with a history of revision [24]. However, revisions of radiological reports may be made for a variety of reasons including insignificant reasons, and the revised words and phrases may be scattered in the context of the report, making it difficult to analyze them. Brigham et al. proposed that radiologists’ self-reported error identification by searching the addenda is another report-focused extraction method [4]; however, this method cannot count error detection by their colleagues. We adopted the keyword search related to “findings visible in retrospect” that could extract the false-negative findings pointed out by self-assessments as well as other radiologists. Using the same keyword search in the context of the reports made it easy to identify the details of false-negative results, as well. Performing our analysis in daily or monthly quality control can be cumbersome and time-consuming. We are currently brushing it up, so that it can be computationally automated.

Our present analyses revealed that the false-negative detection rate during the study period at our hospital was 0.71%, which is lower than 0.8–5% of diagnostic imaging errors in previous studies. This could be due mainly to the possibility that not all findings that are visible in retrospect are necessarily described in the radiological reports. Moreover, physicians are often reluctant to document their colleagues’ mistakes on the record [10]. It would thus be far better to have a common consensus on how to explain false-negative findings. Berlin advised that a report of a misdiagnosis should be succinct, matter-of fact, and nonjudgmental [8]. A simple statement such as “In retrospect, the lesion was present on the radiograph taken January 4, 1993” is sufficient. Words such as “missed,” “error,” and “mistake” and such phrases as “should have been diagnosed” and “was obviously present but not seen” should be avoided [8]. Our low false-negative detection rate might be also affected by the frequency of DR false-negative findings, which was lower than that in previous articles [1, 5]. We only read DRs from selected departments. This is a manifestation of a unique Japanese medical trend to cut DR readings to read numerous CT and MRI examinations [25, 26].

Conventionally, the anatomical classification used in examinations of diagnostic imaging errors has been performed empirically or ad hoc, and not been unified [24]. The anatomical classification is important, because organs, such as lung [27], liver [28], brain [29], and others, have specific regional characteristics that are associated with oversight. The imaging anatomical classification should thus be unified and consistent with the terminology of human anatomy. Our present investigation was the first attempt to re-organize and transfer the anatomical terminology defined by the FIPAT to the new ROC that matches the results of medical imaging.

Most of the organs listed by the FIPAT could be assigned into six ROC categories: head, face–neck, chest, abdomen, extremity, and other. However, the internal, external, and common carotid arteries, the trachea, and the esophagus as boundary organs had to be divided. On the other hand, ‘Pelvis’ in the TA was included in the ROC category Abdomen. This integration allowed us to avoid the vague divisions of the small and large intestines, abdominal vessels, and ureter, which are considered borderline organs between the upper abdomen and pelvis. A problem remains, however: not being able to classify abdominal MR findings precisely, since imaging examinations are usually performed separately for the upper abdomen and pelvis.

The lesion classification of false-negative findings in our study was organized to be completed primarily within the content of the radiological report, so that cases with few definitive diagnoses can be monitored without undue burden. The ROC divides the lesion classification into the six categories of Localized, Vascular, Inflammatory, Traumatic, and Degenerative lesions, Organ dysfunction, and other. These categories may not always be accurate or distinct, but they do not require an excessively profound search for the final diagnosis. Nevertheless, a further re-organization of this classification might be required if new categories that do not belong in the others category are identified.

The most frequent false-negative findings were in Localized lesions in the Lung found on CT, at 22% of all of the false-negative findings detected in this study. This high frequency suggests that a lung lesion must be differentiated from a malignant tumor. If this situation can be ameliorated, 22% of the false-negative results could be suppressed. Until very recently, the practice of diagnostic radiology was solely a human effort, and it was thought that the extermination of false-negative findings could not be achieved [30]. However, artificial intelligence-based computer-assisted diagnosis (AI-CAD) has afforded sensational developments in radiology [31–34].

AI-CAD is computer software that learns image data with labeling and outputs the optimum diagnosis. Although it takes a long time until AI-CAD can catch up with actual human perception [35], some AI-CADs in limited domains are catching up with human imaging-based diagnosis ability. In addition, since various types of AI-CAD are introduced in the future, it is necessary to develop a methodology that enables continuous accuracy analysis by a unified method. Our method for monitoring false-negative findings might be an easy way to verify the performance evaluation of AI-CAD in the near future. Our present analyses identified frequent false-negative findings in localized lung lesions on CT (22%), followed by localized lesions of the liver on CT (6%) and vascular lesions of cerebrovascular disease on MR (5%), and the careful management of these lesions could be the most effective for reducing missed diagnoses, and these results suggest a direction for the development of AI-CAD.

Our study includes some limitations. It is entirely possible that some false-negative findings are not included in radiological reports. However, we observed that the more serious the previously missed findings were in the most current examination, the more likely they were to be mentioned as findings “visible in retrospect” in the current report. This is because the serious findings were usually investigated by multiple physicians. We did not examine whether changes in the number of radiologists during the study period contributed to differences in the number of the false-negative findings. The number of radiologists can influence two factors; the occurrence of the false-negative findings and retrospective detection thereof, which are difficult to simultaneously analyze. Our analysis might not have detected false-negative findings in the one-time-only examinations performed at our hospital due to a lack of observation of temporal changes. Our study may have failed to detect the false-negative findings that would have been detected if multiple radiologists made careful observations. There could also be findings that one radiologist believes to be a false negative, while another radiologist believes them to be a true negative. The “visible in retrospect”-related keywords used in our search may not have identified all false-negative findings. In terms of being able to complete with a radiological report, however, our method is easy to use as a surrogate monitoring system for false-negative findings, even if it is not perfect.

## Conclusions

Our analysis revealed regional and lesion characteristics for false-negative findings in the whole body across a wide variety of imaging modalities. Our results demonstrated that missed lung localized lesions on CT, which account for about a fifth of false-negative findings, were the most common false-negative finding.

## Data Availability

Please specify the source when disclosing this data. This data cannot be used for commercial purposes. If you modify this data, please specify the procedure by some means.

## Appendix

### The principal points for matching general anatomy in the TA and the major ROC categories

1. ‘Neurocranial part of head’ in the Head category of the TA was assigned as ‘Head’ in the ROC.
2. The ‘Face’ part in Head of the TA was separated and assigned in combination with ‘Neck’ of the TA as ‘Face-Neck’ in the ROC.
3. ‘Thorax,’ ‘Anterolateral thoracic wall,’ ‘Back of thorax’ in ‘Trunk’ of the TA were assigned to the Chest category of the ROC. ‘Shoulder’ and ‘Axilla,’ including ‘Scapula’ and ‘Clavicle,’ in 'Upper limb' of the TA were also assigned to Chest in the ROC.
4. ‘Abdomen,’ ‘Pelvis,’ ‘Anterolateral abdominal wall,’ and ‘Back of abdomen’ in the TA Trunk category were assigned to the ROC category ‘Abdomen.’ ‘Buttock’ and ‘Hip’ in ‘Lower limb' of the TA were also assigned to ‘Abdomen’ in the ROC.
5. ‘Free part of upper limb’ and ‘Free part of lower limb’ as categorized in the TA were assigned to ‘Extremity’ in the ROC.
6. We assigned false-negative findings that were across multiple regions to the predominant region of the lesion distribution, but a false-negative finding extending equally to multiple regions was assigned to the ‘Other’ category of the ROC, especially concerning whole-body bone.

### Boundaries between major categories of the ROC

Accordingly, five boundaries between the six major ROC categories were defined as follows (Fig. 1): (1) between the head and the face–neck: The lower margin of the skull base, which is approximately consistent with the supra-orbito-meatal line. (2) Between the face–neck and the chest: the manubrium, the upper margin of first ribs, and the midplane of 7th cervical—1st thoracic vertebral (C7/T1) intervertebral disc. (3) Between the chest and abdomen: the diaphragm and the midplane of the 12th thoracic—1st lumbar vertebral (T12/L1) intervertebral disc. (4) Between the chest and an upper extremity: the shoulder joint. (5) Between the abdomen and a lower extremity: the hip joint.

### Details of matching the TA to the ROC

Some organs overlapped in more than one regional category, and we thus divided them into two or three parts and assigned them to the proper parts of the ROC as follows:

1. ‘Esophagus’ in the TAs’ Chapter 5 (Digestive System): we divided the esophagus into three parts, i.e., the cervical, thoracic, and abdominal esophagus, which were assigned to Face–Neck, Chest, and Abdomen, respectively.
2. ‘Trachea’ in the TA’s Chapter 6 (Respiratory system): the ROC divided the trachea into two parts, i.e., the cervical and mediastinal trachea, which were assigned to Face–neck and Chest, respectively in the ROC.
3. ‘Internal carotid artery’ (ICA), ‘External carotid artery’ (ECA), and ‘Vertebral artery’ (VA) in the TA’s Chapter 13 (Cardiovascular System); they were divided into two parts at the above-mentioned regional boundary, with each vessel’s upper part assigned to Head and the lower part assigned to Face-Neck.
4. ‘Left Common carotid artery’ (CCA) in the TA’s Chapter 13 (Cardiovascular System); the ROC divided left CCA into two parts by the above-mentioned regional boundary into the superior part and inferior part of each of these arteries assigned to Face–neck and Chest, respectively. ‘Right common carotid artery’ (RCA) was assigned to Face–Neck in the ROC.
5. ‘Brachiocephalic artery’ and ‘Subclavian artery’ in the TA’s Chapter 13 (Cardiovascular System); they were assigned to the Chest category of the ROC.
6. Integument was included in bone & soft tissue. As an exception, ‘Breast’ in the TA’s ‘Integument’ category was assigned as an organ of Chest in the ROC.

All organs in Chapters 2 to 16 in the TA other than those described above, i.e., Bones, Joints, Muscular System, Digestive System, Respiratory System, Thoracic Cavity, Urinary System, Genital Systems, Abdominopelvic Cavity, Endocrine Glands, Cardiovascular System, Lymphoid Organs, Nervous System, Sense Organs, and Integument, were separated according to above-mentioned boundaries for the ROC and re-classified in the six major ROC categories. For example, the following TA-to-ROC assignments were made for the TA category ‘Endocrine Glands’: Hypophysis, Head; ‘Pineal gland’, Head; ‘Thyroid gland’, Face–Neck; ‘Parathyroid glands’, Face–Neck; and ‘Suprarenal (Adrenal) gland’, Abdomen.

## Abbreviations

CT: Computed tomography
MR: Magnetic resonance imaging
FDG-PET-CT: Positron emission tomography and computed tomography using ^18^F-fluorodeoxyglucose
DR: Digital radiography
NM: Nuclear medicine
TA: Terminologia Anatomica
FIPAT: The Federative International Programme for Anatomical Terminology
ROC: Regional organ classification

## References

[1] Berlin L (2014). Radiologic errors, past, present and future. Diagnosis (Berlin, Germany).

[2] Brady A, Laoide RO, McCarthy P, McDermott R (2012). Discrepancy and error in radiology: concepts, causes and consequences. Ulst Med J.

[3] Waite S, Grigorian A, Alexander RG, Macknik SL, Carrasco M, Heeger DJ (2019). Analysis of perceptual expertise in radiology—current knowledge and a new perspective. Front Hum Neurosci.

[4] Brigham LR, Mansouri M, Abujudeh HH (2015). JOURNAL CLUB: radiology report addenda: a self-report approach to error identification, quantification, and classification. AJR Am J Roentgenol.

[5] Kim YW, Mansfield LT (2014). Fool me twice: delayed diagnoses in radiology with emphasis on perpetuated errors. AJR Am J Roentgenol.

[6] Federative International Programme for Anatomical Terminology (FIPAT). Terminologia Anatomica. 2nd ed. International federation of associations of anatomists (IFAA). 2019. https://fipat.library.dal.ca/ta2/ Accessed 7 May 2022.

[7] Renfrew DL, Franken EA, Berbaum KS, Weigelt FH, Abu-Yousef MM (1992). Error in radiology: classification and lessons in 182 cases presented at a problem case conference. Radiology.

[8] Berlin L (1994). Reporting the "missed" radiologic diagnosis: medicolegal and ethical considerations. Radiology.

[9] Simmons MZ (1995). Reporting "missed" radiologic diagnoses. Radiology.

[10] Lee JK (2007). Quality–a radiology imperative: interpretation accuracy and pertinence. J Am Coll Radiol.

[11] Taylor GA, Voss SD, Melvin PR, Graham DA (2011). Diagnostic errors in pediatric radiology. Pediatr Radiol.

[12] Hawkesford MPH, Kalogrianitis S (2015). Delayed diagnosis of lung cancer after missed vertebral metastasis on CT. BJR case reports.

[13] Brook OR, O'Connell AM, Thornton E, Eisenberg RL, Mendiratta-Lala M, Kruskal JB (2010). Quality initiatives: anatomy and pathophysiology of errors occurring in clinical radiology practice. Radiographics.

[14] Alpert HR, Hillman BJ (2004). Quality and variability in diagnostic radiology. J Am Coll Radiol.

[15] Schiff GD, Hasan O, Kim S, Abrams R, Cosby K, Lambert BL (2009). Diagnostic error in medicine: analysis of 583 physician-reported errors. Arch Intern Med.

[16] Heriot GS, McKelvie P, Pitman AG (2009). Diagnostic errors in patients dying in hospital: radiology's contribution. J Med Imaging Radiat Oncol.

[17] Chin SC, Weir-McCall JR, Yeap PM, White RD, Budak MJ, Duncan G (2017). Evidence-based anatomical review areas derived from systematic analysis of cases from a radiological departmental discrepancy meeting. Clin Radiol.

[18] Donald JJ, Barnard SA (2012). Common patterns in 558 diagnostic radiology errors. J Med Imaging Radiat Oncol.

[19] Lauritzen PM, Andersen JG, Stokke MV, Tennstrand AL, Aamodt R, Heggelund T (2016). Radiologist-initiated double reading of abdominal CT: retrospective analysis of the clinical importance of changes to radiology reports. BMJ Qual Saf.

[20] Lauritzen PM, Stavem K, Andersen JG, Stokke MV, Tennstrand AL, Bjerke G (2016). Double reading of current chest CT examinations: clinical importance of changes to radiology reports. Eur J Radiol.

[21] Patel SH, Stanton CL, Miller SG, Patrie JT, Itri JN, Shepherd TM (2019). Risk factors for perceptual-versus-interpretative errors in diagnostic neuroradiology. AJNR Am J Neuroradiol.

[22] Carrara M, Yakar D, Kasalak O, Kwee TC (2020). A new complication registration system for errors in radiology: Initial 5-year experience in a tertiary care radiology department. Eur J Radiol.

[23] Kielar AZ, McInnes M, Quan M, O'Sullivan J (2011). Introduction of QUIP (quality information program) as a semi-automated quality assessment endeavor allowing retrospective review of errors in cross-sectional abdominal imaging. Acad Radiol.

[24] Diaz S, Ekberg O (2010). The frequency of diagnostic errors in radiologic reports depends on the patient's age. Acta Radiol.

[25] Nishie A, Kakihara D, Nojo T, Nakamura K, Kuribayashi S, Kadoya M (2015). Current radiologist workload and the shortages in Japan: how many full-time radiologists are required?. Jpn J Radiol.

[26] Nakajima Y, Yamada K, Imamura K, Kobayashi K (2008). Radiologist supply and workload: international comparison–working group of Japanese college of radiology. Radiat Med.

[27] Chen H, Huang S, Zeng Q, Zhang M, Ni Z, Li X (2019). A retrospective study analyzing missed diagnosis of lung metastases at their early stages on computed tomography. J Thorac Dis.

[28] Nakai H, Arizono S, Isoda H, Togashi K (2019). Imaging characteristics of liver metastases overlooked at contrast-enhanced CT. AJR Am J Roentgenol.

[29] Davis PC, Hudgins PA, Peterman SB, Hoffman JC (1991). Diagnosis of cerebral metastases: double-dose delayed CT vs contrast-enhanced MR imaging. AJNR Am J Neuroradiol.

[30] Bruno MA (2017). 256 Shades of gray: uncertainty and diagnostic error in radiology. Diagnosis (Berlin, Germany).

[31] Krizhevsky A, Sutskever I, Hinton GE. ImageNet classification with deep convolutional neural networks. Proceedings of the 25th international conference on neural information processing systems—Volume 1; Lake Tahoe, Nevada. 2999257: Curran Associates Inc.; 2012; p. 1097–105.

[32] Noguchi T, Higa D, Asada T, Kawata Y, Machitori A, Shida Y (2018). Artificial intelligence using neural network architecture for radiology (AINNAR): classification of MR imaging sequences. Jpn J Radiol.

[33] Noguchi T, Uchiyama F, Kawata Y, Machitori A, Shida Y, Okafuji T (2020). A fundamental study assessing the diagnostic performance of deep learning for a brain metastasis detection task. Magn Reson Med Sci.

[34] Nakata N (2019). Recent technical development of artificial intelligence for diagnostic medical imaging. Jpn J Radiol.

[35] Yamada K, Mori S (2019). The day when computers read between lines. Jpn J Radiol.

